# Reliability and validity of new isokinetic strength assessment for rotator cuff muscles in a muscle architecture-based position

**DOI:** 10.55730/1300-0144.5774

**Published:** 2023-11-25

**Authors:** Çağlar SOYLU, Mustafa Ertuğrul YAŞA, Pervin DEMİR, Ahmet Mustafa ADA, Tüzün FIRAT, Necmiye ÜN YILDIRIM

**Affiliations:** 1 Department of Physical Therapy and Rehabilitation, Gülhane Faculty of Physical Therapy and Rehabilitation, University of Health Sciences, Ankara, Turkiye; 2 Department of Basic Medical Sciences, Biostatistics and Medical Informatics, Faculty of Medicine, Ankara Yıldırım Beyazıt University, Ankara, Turkiye; 3 Turkish Armed Forces Sport School, Physical Fitness Test and Evaluation, Ankara, Turkiye; 4 Department of Physical Therapy and Rehabilitation, Faculty of Physical Therapy and Rehabilitation, Hacettepe University, Ankara, Turkiye

**Keywords:** Muscle architecture, rotator cuff, muscle strength dynamometer, reproducibility of results

## Abstract

**Background/aim:**

Isokinetic strength assessment of the rotator cuff muscle is frequently applied in a variety of shoulder postures, but none of these consider muscular architecture, which is one of the most important aspects of improving strength development. This study aimed to examine the test and retest reliability and validity of the muscle architecture-based position (MABP), which is 25° abduction and 20° external rotation, in healthy subjects to be able to select a better isokinetic assessment position for shoulder rotator cuff muscles.

**Materials and methods:**

A total of 54 healthy males with a mean age of 21.0 ± 1.2 years and mean body mass index of 22.8 ± 1.7 kg/m^2^ completed an isokinetic measurement session. All of the tests were performed on an IsoMed 2000 isokinetic dynamometer concentrically and eccentrically for both upper limbs at 60°/s angular velocity. All of the participants completed 3 measurement sessions: the first represented the isokinetic testing and was performed in the scapular neutral position (SNP) (45° shoulder flexion and abduction), the second represented the MABP (25° abduction and 20° ER) for shoulder rotator cuff muscles, and the third represented the test and retest of the MABP.

**Results:**

The correlations between the 2 techniques for assessing concurrent validity ranged from 0.908 to 0.994. The values obtained from the MABP were higher than those obtained in the SNP. There was no systematic bias for any measurements between the MABP and the retest of the MABP (p > 0.05). The intraclass correlation coefficients representing the test and retest reliability results for each variable measured with the MABP was higher than 0.98 and this value was considered as excellent reliability.

**Conclusion:**

In conclusion, the MABP can be used to assess the isokinetic strength of the rotator cuff muscles safely and confidently, with increased quantities of force being released and measurement at optimal muscle tension.

## 1. Introduction

All body motions occur through the contraction of a muscle that can maintain its contractile characteristic. Muscles are the only tissues capable of producing force for movement or stabilization. In this regard, one might argue that strength is the most significant measure of a healthy muscle [1]. Many different methods and systems for assessing muscle strength have been established over the years. Among these, manual muscle testing has been used for many years by researchers and clinicians as it is quick and simple to apply, and it has maintained its popularity despite technological advancements [2]. However, hand-held dynamometers (break test) are favored over system-based manual muscle testing as a more thorough analysis can be made [3]. In the last quarter-century, medical technological advances have culminated in the implementation of more objective, detailed, and effective methods of assessment and care. Isokinetic dynamometers are the gold standard equipment with current technological options which can evaluate in detail and objectively each of the parameters of the muscle dynamic neuromuscular performance [4].

The relationship between the sarcomere length and strength in skeletal muscle has been studied for many years and is a well-defined phenomenon. Lieber et al. examined the amount of force produced by the wrist extensor muscles, between the sarcomere lengths of 1.6 μm (maximum actin and myosin interaction) and 3.6 μm (minimum actin and myosin interaction). Accordingly, the interval in which the sarcomere length was 2.6–2.8 μm was determined as the maximum actin-myosin interaction and the highest force. This measurement refers to the optimal sarcomere length for producing maximum force [5,6].

In isokinetic measurements, the test position chosen is the most important factor in determining the result, and the repeatability of the measurements is directly related to the position chosen [7]. To date, various studies have examined the amount of isokinetic force produced by the rotator cuff muscles in different joint positions in the shoulder joint, which offers a wide variety of test positions and joint planes for isokinetic evaluation due to its relatively wide freedom of movement [7–9]. However, comparisons between studies are difficult due to a lack of standardization in the protocols used and the use of different device brands. According to Forthomme et al., the most accurate and reproducible shoulder internal rotation (IR) and external rotation (ER) strength test position was 90° or 45° arm abduction in the frontal plane when lying supine [8]. The most popular isokinetic test position for the rotator cuff muscles today is the scapular plane, which is recognized as the most practical position for the rotator cuff muscles [9]. However, considering the architectural characteristics of the rotator muscles, the suitability of a position in which the optimum sarcomere length is obtained has not yet been investigated. At the conclusion of research on the rotator cuff muscles architecture, Ward et al. stated that the rotator cuff muscles collectively achieve maximal force-producing potential at approximately 25° abduction and 20° ER, with the sarcomere length of the muscles in the range of 2.6–2.8 μm [6]. Since the optimal sarcomere length is a major factor for the optimal force that the muscle can produce, the shoulder position that they defined is a determining factor for the force capacity of the rotator cuff muscles. The purpose of this study was to examine the test and retest reliability and validity of the muscle architecture-based position (MABP), which is 25° abduction and 20° ER. It was hypothesized herein that the MABP position is a valid and reliable method and that the values obtained from the MABP would be higher than those obtained from the scapular neutral position (SNP).

## 2. Methods

### 2.1. Participants

To determine the study sample size, 2 different calculations were made according to the study design using the preliminary results. G-Power Version 3.1.9.2 [10] and the Intraclass correlation coefficient (ICC) Sample size package [11] in R language [12] were used to calculate the required sample size.

The ICC value from the preliminary study was determined as a minimum 0.962 for eccentric (ECC) 60°/s IR nondominant side (NDM) peak torque (PT) (ECC 60 IR NDM PT) and ECC 60 IR NDM PT/weight (W) variables. The required minimum sample size to determine the agreement between 2 measurements of the MABP was obtained as 33 based on r_0_: 0.75 (lower bound for good reliability), r: 0.90 (lower bound for excellent reliability), 0.80 of power, and type I error of 0.05 (54 for the power of 0.95).

All of the study participants were healthy and met the following inclusion criteria: 1) aged 18–35 years, and 2) no history of injury or surgery that may affect the upper limbs. Participants were excluded if 1) they had upper extremity injuries that had limited their physical activity level in the past 3 months, 2) they had surgery of the lower and/or upper extremity in the previous one year, 3) they had a diagnosis (pain, disability, instability, limited range of motion (ROM) in shoulder function, chronic disease, etc.) that could influence their strength ability, or 4) they were a professional level athlete. A total of 54 healthy males with a mean age of 21.0 ± 1.2 years and mean body mass index (BMI) of 22.8 ± 1.7 kg/m^2^ completed an isokinetic measurement session.

All of the participants agreed to take part in the study and gave written informed consent. The study was approved by the university Ethics Committee (registration number 2019/367) and was conducted in accordance with the Declaration of Helsinki, registered at ClinicalTrials.gov (NCT 04755257).

### 2.2. Procedure

**Figure 1:**
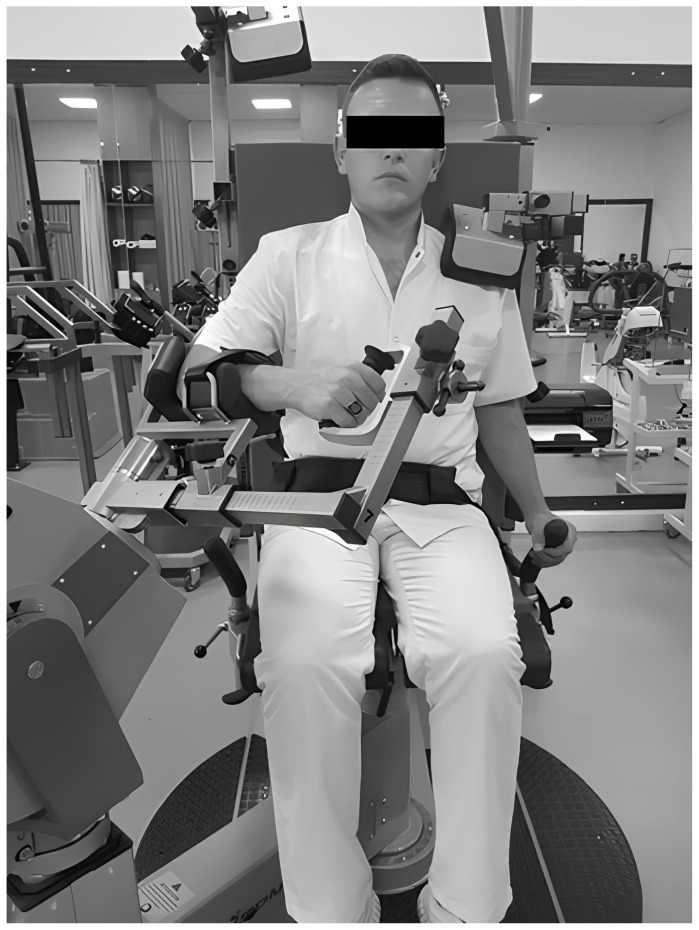
SNP (45° of shoulder abduction in the scapular plane).

**Figure 2:**
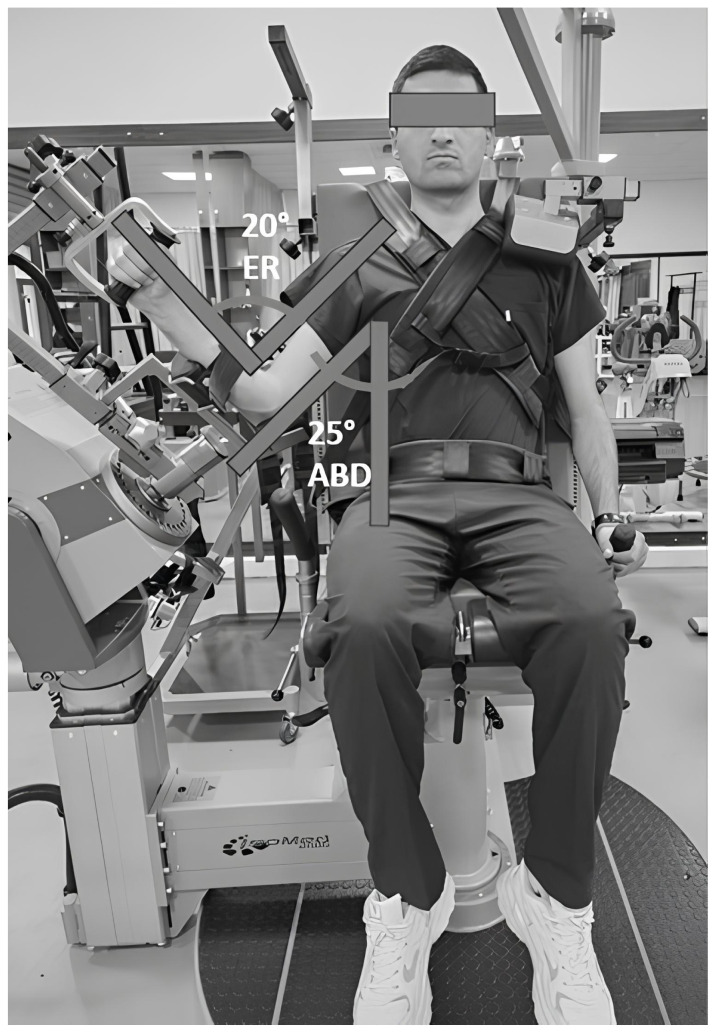
MABP (25° abduction and 20° ER).

A repeated-measurement design was used to evaluate the shoulder rotator strength with different protocols. All of the tests were performed on an IsoMed 2000 isokinetic dynamometer (D. & R. Ferstl GmbH, Hemau, Germany). All of the participants completed 3 measurement sessions: the first represented the isokinetic testing and was performed in the scapular neutral position (SNP) (45° shoulder flexion and abduction) (Figure. 1), the second represented the MABP (25° abduction and 20° ER) for shoulder rotator cuff muscles (Figure. 2), and the third represented the test and retest of MABP. Test and retest was applied to the same participants 7–14 days later by the same rater at the same time of day [13]. All of the evaluations were conducted for each participant under similar environmental conditions (~21 °C and ~60% humidity). The measurement positions were performed in randomized order to eliminate the learning effect. The same order of testing was followed in the other sessions and all of the volunteers were advised to maintain their normal level of physical activities between testing sessions.

Before taking the isokinetic tests, all of the participants had a 5-min warmup period in an arm crank ergometer at a slow tempo followed by 3–5 min of stretching exercises [13,14]. The participants were then taken to the isokinetic device to be measured individually, and the device was calibrated in accordance with the anthropometric structures of each participant. During the test, the height, weight, date of birth, and dominant upper extremity values of the participants were entered into the computer program. The dominant shoulder was defined as the hand used for writing. Before starting the test, verbal instructions were given to push as hard and fast as possible through the full ROM. During the tests, verbal commands were given with the same tone in each movement to guide and motivate each individual. The participants were seated in an upright position, as described in the user manual, with the backrest at 80° [13,15].

#### Isokinetic measurement positions

##### SNP

Edouard et al. reported that the seated position with 45° of shoulder abduction in the scapular plane seemed the most reliable for IR and ER strength assessment [13]. Therefore, this position was used for comparison, validity, and reliability with the newly developed MABP position. Participants were seated with the shoulder in 45° of abduction, and the elbow flexed to 90° (Figure.1). The joint angles required for both positions were adjusted with a J-Tech dual inclinometer (J-Tech Medical, Midvale, UT, USA).

##### MABP

The sarcomere length was not measured for the MABP. Reference angle values recommended by Ward et al. were used. They suggested that the rotator cuff muscles collectively reach their optimum force-producing capacity at approximately 25° abduction and 20° ER, which also means the position of optimal sarcomere length for rotator cuff muscles [6]. Therefore, this position was used to develop a new isokinetic measurement position for shoulder rotator muscles. The participants were seated in an upright position, with the shoulder in 25° of abduction and 20° ER, and the elbow flexed to 90° (Figure.2). The joint angles required for both positions were adjusted with the J-Tech dual inclinometer.

In both measuring positions, the elbow was placed in the elbow stabilizer pad and fixed with a Velcro strap, so that the humeral shaft (i.e. shoulder axis of rotation) was in line with the axis of rotation of the dynamometer. To minimize trunk movement, the trunk was secured with stabilizing straps and a Velcro strap was placed over the iliac crest. At the same time, the effect of gravity has been reset. The ROM of the test was between 90° IR and 0° ER for both methods. After each measurement, the participants were asked whether they felt any discomfort. It was stated by the participants that there was no discomfort in any of the test sections.

#### Test protocol

For familiarization with the IsoMed 2000 and the test procedure, the participants performed 3 submaximal IR and ER rotation movement trial repetitions at 60°/s angular velocity prior to each test. After the warmup period and a 30-s interval, maximal IR/ER rotation movement was repeated 5 times concentrically and eccentrically for both upper limbs at 60°/s angular velocity. A 30-s rest period was offered between trials and the test, and a 2-min rest interval after each test. The PT and PT/W values for the concentric (CON) contraction and the ECC contraction in both methods were recorded for each participant [16].

### 2.3. Statistical analyses

The Shapiro–Wilk test and the coefficient of variation were applied to check the normal distribution assumption of continuous variables. The variables were reported adopting descriptive statistics (mean ± standard deviation, minimum, maximum, median with 95% confidence interval (CI) based on quantiles of the binomial distribution).

Validity: To verify if there were any systematic differences between the results of the 2 tests, the Wilcoxon signed-rank test was applied. Spearman’s rank correlation was calculated to determine the relationship between the 2 methods (equal to the ICC calculated from the ranks) to assess concurrent validity. The results were interpreted as 0–0.29 negligible, 0.30–0.49 low, 0.50–0.69 moderate, 0.70–0.89 high, and 0.90–1.00 very high correlation [17].

Reliability: The same rater performed the test and retest procedure with a single participant. The Wilcoxon signed-rank test was applied to determine any systematic bias between the test and retest results. Reliability was determined by calculating the ICC, single measure, 2-way mixed-effects model, where people effects are random and measure effects are fixed, absolute agreement) and the 95% CI. The results were interpreted as <0.50: poor reliability, 0.50–0.75: moderate reliability, 0.76–0.90: good reliability, and >0.90: excellent reliability [18].

The standard error of measurement (SEM = SD √ (1-ICC), with SD representing the standard deviation of the measure) was calculated to determine the amount of variation in the measurement errors for the new method [19]. The smallest detectable difference (SDD), the change in the measurement score beyond the measurement error, was calculated as follows: SEM × 1.96 × √2. The SDD% was calculated as ([SDD/grand mean] × 100), where the grand mean represents the mean score of all of the trials.

The difference between the test and retest scores was not distributed normally. Therefore, the quantile estimations based on order statistics [20] were used to create the Bland-Altman graph, which was used to show the magnitude of the difference of repeated measurements, showing the difference between test and retest (y-axis) against the mean of the 2 measurements (x-axis) [21]. The median and nonparametric upper and lower limits of agreement (LoA), which span 95% of the observations, were determined from the values in the sample of the 50th, 97.5th, and 2.5th percentiles, respectively. The 95% CIs for the percentiles were calculated based on the quantiles of the binomial distribution.

The statistical analyses were performed using IBM SPSS Statistics for Windows 21.0 (IBM Corp., Armonk, NY, USA) and R software DescTools package to calculate the ICCs and CIs of the quantiles [22]. the RVAideMemoire package was used to calculate Spearman coefficient and CIs [23], and the ggplot2 package was used to draw the graph for the 95% CI of the median [24]. The pandas [25] and matplotlib [26] packages in Python3 software [27] were used to draw the Bland-Altman plots. Statistical significance was accepted as 2-sided p ≤ 0.05.

## 3. Results

The mean age of the study sample was 21.0 ± 1.2 years (median: 21, 95% CI of the median: 20–21, min: 20, max: 25) and the mean BMI was 22.8 ± 1.7 kg/m^2^ (median: 23.4, 95% CI of the median: 22.6–23.7, min: 18.6, max: 26.1).

The descriptive statistics for the SNP, MABP and their differences are summarized in Table 1. A statistically significant difference was determined between the 2 methods (p < 0.001). When the descriptive statistics and systematic differences were examined for all of the measurements, the values obtained from the MABP were higher than those obtained in the SNP. The correlations between the 2 methods used to evaluate the concurrent validity were very high for all of the variables. The correlations ranged from 0.908 to 0.994.

**Figure 3:**
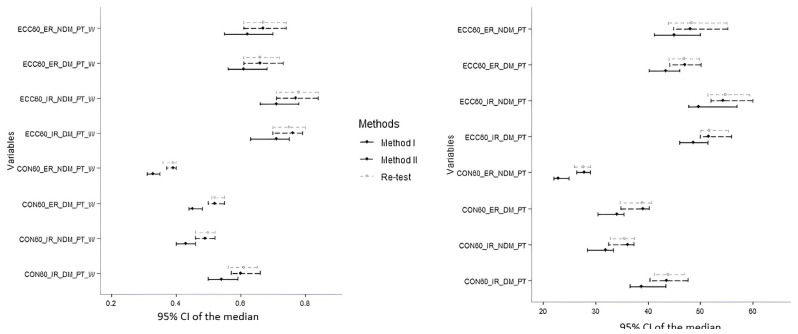
Median and 95% CI of the median for all measurements obtained from the SNP, MABP, and retest (there was a significant systematic difference between the 2 methods, but there was no statistically significant difference between test and retest measurements of the MABP).

To represent the differences and similarities between the SNP, MABP, and retest of the MABP, the median and 95% CI graphs were drawn for each measurement. There was a significant systematic difference between the 2 methods, but there was no statistically significant difference between test and retest measurement of the MABP (Figure 3).

**Figure 4:**
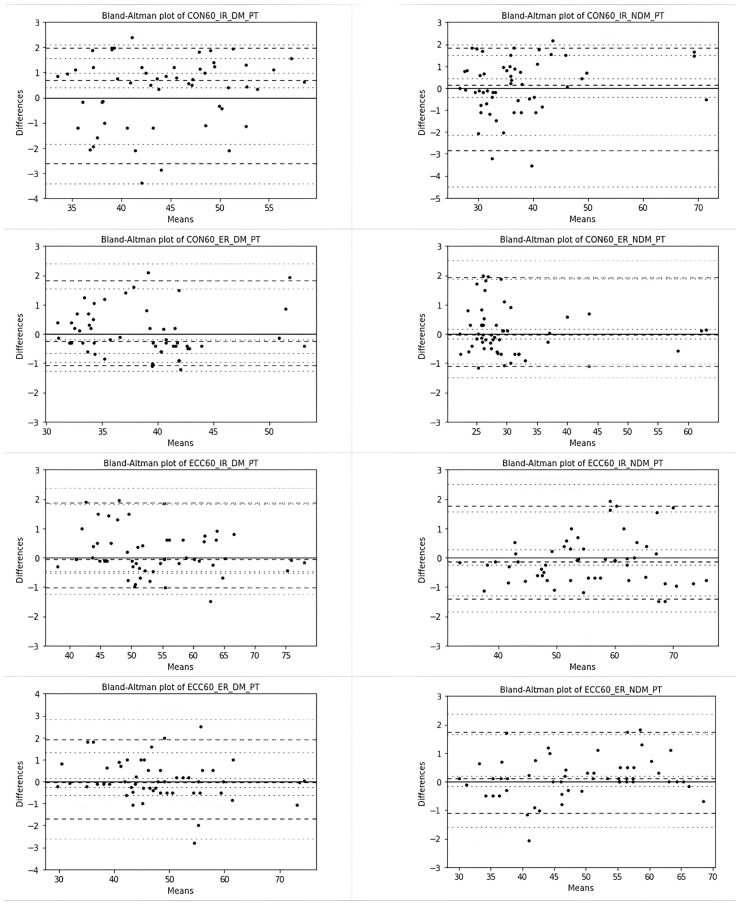
Bland-Altman plots for the test and retest reliability of the PT measurements based on nonparametric quantile estimators (the dashed lines represent the nonparametric limits of agreement and median of differences, while the dotted lines represent 95% CI based on binomial distribution).

**Figure 5:**
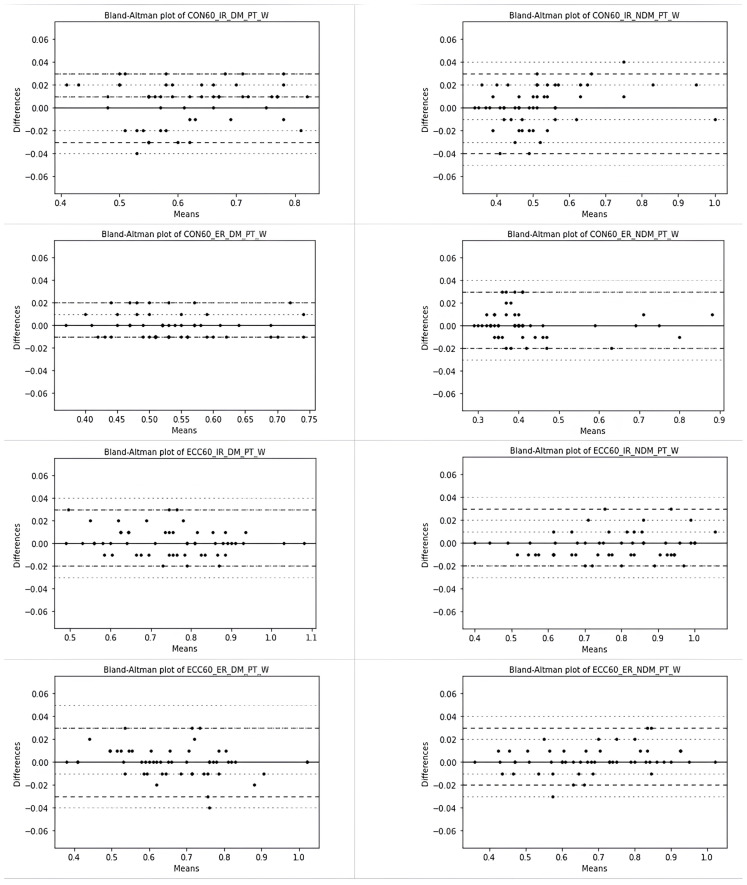
Bland-Altman plots for the test and retest reliability of the PT_W measurements based on nonparametric quantile estimators (the dashed lines represent the nonparametric limits of agreement and median of differences, while the dotted lines represent 95% CI based on binomial distribution).

The systematic and random errors for the MABP are summarized in Table 2 and Figures 4 and 5. There was no systematic bias for any of the measurements between the MABP and the retest of the MABP (p > 0.05). The ICC value, representing the test and retest reliability results for each variable measured with the MABP was higher than 0.98 and this value was considered as excellent reliability. The SEM values were 0.01 for the PT_W measurements and ranged from 0.52 to 0.98 for the PT measurements. The lowest and upper SDD% values used to compare the test and retest reliability among the measurements were obtained for the ECC 60 IR DM PT/W measurement (2.84) and the CON 60 IR NDM PT/W measurement (6.57).

The random error was very low, especially for the PT_W values. The 2.5% and 97.5% percentiles, as the nonparametric limits of agreement, are expected to lie within the range defining 95% of all of the observed differences between the test and retest measurements, and these are shown with Bland-Altman graphs in Figures 2 and 3. They showed no systematic trend. Bias values that deviated from the nonparametric LoA limits were extremely rare.

## 4. Discussion

This study, which was conducted to determine a new isokinetic strength test position for the shoulder rotator muscles, is the first in the literature to investigate the validity and reliability of the isokinetic measurement made in the shoulder position, where the optimum sarcomere length was obtained based on the architectural features of the muscles. The results of this investigation showed excellent reliability, with ICC values >0.98 and validity with r values >0.91, which means very high. In addition, the rotator cuff muscles produced more power when tested in the MABP. These findings confirm that the MABP for shoulder rotator cuff muscles, which is 25° abduction and 20° ER, is a reliable and valid test position for assessing shoulder rotation strength in healthy male adults.

The architectural arrangement of the rotator cuff muscle fibers implies that they are built for force production rather than excursion, which is compatible with their suggested function of glenoid stabilization. Therefore, rotator cuff muscles (especially the supraspinatus) are extremely sensitive to changes in length and function [6]. When focusing on rotator muscle strength assessment, the shoulder positioning during the test influences the strength developed. According to several studies, isokinetic shoulder evaluation in the position of 30° to 45° shoulder abduction of the shoulder joint in the scapular plane is a more natural and functional movement than movements in the sagittal or frontal plane [13]. However, the sarcomere length-joint angle relationship of the rotator cuff muscles is not considered in this measurement position. The only approach for determining relative muscle force as a function of joint angle is the sarcomere length-joint angle relationship (operating range). This assessment has been used to forecast the impact of rehabilitative therapies as well as provide information about normal muscle function [6]. It was reported that in order for a muscle to produce maximum performance, it should have an optimal sarcomere length in the range of 2.6–2.8 μm [5,6]. Ward et al. stated that this optimal sarcomere length for the rotator cuff muscles corresponds to a position of approximately 25° abduction and 20° ER, which has optimal force generation capacity [6]. In addition, when rotator cuff injury mechanisms were examined, the decrease in the force-generating capacity due to the changing length-tension relationship was accepted as an important reason [28,29]. As the sarcomere length plays a key role in determining the length-tension relationship, it is seen that the passive tension of the rotator cuff muscles in the 30° to 45° shoulder abduction position in the scapular plane suggested in the literature is high and deviates from the optimal sarcomere position [6,29]. It was found herein that this position, which was stated as more natural and functional, was actually weaker in the isokinetic evaluation of the rotator cuff muscles compared to the MABP was developed in this study. Thus, these results suggest that the MABP may be a more desirable position for assessing and rehabilitating the shoulder-rotator muscles.

Selecting the angular velocity represents another cornerstone in isokinetic measurement methodology [8]. Dvir stated that there was no standardization in the current angular velocity selection. As the primary purpose of isokinetic testing is the assessment of bilateral strength differences, he suggested that any velocity which is well tolerated by individuals could serve for this particular purpose [30]. However, in recent studies, a 60°/s angular velocity has been frequently used and recommended for isokinetic strength assessment of shoulder IR and ER muscles [15,31,32]. Forthomme et al. [8] and Durall et al. [33] also reported higher reproducibility of PT measurement of the rotator cuff muscles in the scapular plane at an angular velocity of 60°/s. Therefore, in the current study, a 60°/s angular velocity was selected for the strength test of the rotator cuff muscles, in accordance with the literature. When the contraction mode was examined, which is another important parameter in the isokinetic evaluation, the shoulder rotator muscle strength was evaluated in 2 modes as ECC and CON. However, Forthomme et al. evaluated shoulder rotator strength only in ECC mode [8]. Nevertheless, the ECC mode would be of definite relevance in an athletic population assessment [34]. In line with other studies, concerning the mode of contraction, both ECC and CON modes were measured [9,13,35,36], because it was stated that the strength deficits in both modes and/or the strength asymmetry between both sides predispose the rotator cuff to injuries [37].

In order for a measurement method to be used in scientific practice, it is necessary to compare it with other confirmed methods. Therefore, making a comparison with the results of the frequently used and trusted methods provides data about the concurrent validity of the new method. In this study, concurrent validity was ensured by examining the correlation between the rank values of the individuals, and systematic difference between the measurement results in the SNP, which is frequently used in measurement routine and the MABP. The concurrent validity values obtained were fairly high (r, such as rank ICC > 0.91). When the median values and CIs were examined, it was determined that the MABP values were higher. These results showed that although higher values were obtained with the MABP method, it was also compatible with the SNP method in terms of the rank values.

Nevertheless, it is critical that the outcomes of a measurement method are both valid and trustworthy. To this end, the MABP test and retest reliability was examined in addition to whether there were any systematic or random errors, and the test and retest reliability was determined to be quite strong, and the values between the 2 methods were similar (ICC > 0.98). In particular, the lack of a systematic trend in the Bland Altman graphs for the PT_W values demonstrated that there was no random error. The systematic error values between the 2 methods were also quite low in the PT_W measurements. Therefore, for more accurate comparison and measurement results, PT_W values should be considered and recommended rather than PT values, as suggested in other isokinetic studies.

Although the isokinetic strength test is a powerful technique for obtaining objective data on shoulder strength following rotator cuff repair, it is not without its limitations. When employed in patients after rotator cuff restoration, however, it was noted that some subjects, unlike healthy individuals, were unable to place and retain the operated arm in the test position [38]. The strengths of this study were as follows: the position had a significant effect during ECC ER and IR and CON ER and IR. In all of the instances when the PT was significant, the PT values were larger in the MABP position compared to the SNP position. The new test position presented in this study has the strongest advantage of providing adequate sarcomere length for the rotator cuff muscles, which can prevent excessive muscle tensions that can affect surgical repair in isokinetic measurements. Further studies are needed to investigate the effectiveness of the MABP in patients following rotator cuff repair.

This study also had some limitations. This research was conducted on healthy males who were free of any illness. Further studies on various demographic groups (women, sports-specific athletes, patient groups) will enable greater understanding of the various possibilities that this new test position can offer. In addition, some of the limitations noted by Ward may also apply to this study. For example, the muscle excursion functions were regression-based, and regional sarcomere differences were not considered in that study. Thus, these variations were calculated based on the regression-based values. These angles may be questionable, so the angles suggested here may not always have a clinical counterpart.

## 5. Conclusion

The functionality of rotator cuff muscles with relatively shorter fiber lengths is extremely sensitive to length variations. This reveals the importance of providing the optimal sarcomere length for accurate and precise results to be obtained from the measurements to be made. According to the findings of this study, the MABP measurement concentrically and eccentrically for both upper limbs at 60°/s angular velocity was found to be valid and reliable in healthy male adults. Therefore, the MABP can be utilized safely and confidently in the isokinetic strength measurement of the rotator cuff muscles, with higher amounts of force being released and measurement at optimal muscle tension.

## Figures and Tables

**Table 1:** Comparisons of differences between the SNP and MABP (n = 54).

Measurements	SNP		MABP		Difference (MABP – SNP)*		r_s_ (95% CI)
	Mean ± SD	Median (min, max)	Mean ± SD	Median (min, max)	Mean ± SD	Median (min, max)	
CON 60 IR DM PT (N/m)	40.21 ± 6.20	38.70 (30.60, 54.10)	44.50 ± 6.47	43.60 (34.00, 58.85)	4.29 ± 1.49	4.70 (1.30, 6.70)	0.968 (0.936, 0.978)
CON 60 IR NDM PT (N/m)	33.43 ± 9.39	31.85 (24.10, 66.30)	37.43 ± 9.81	36.05 (26.70, 71.12)	4.00 ± 1.25	4.38 (1.30, 6.40)	0.956 (0.909, 0.975)
CON 60 IR DM PT/W (N/m.kg)	0.56 ± 0.09	0.54 (0.38, 0.75)	0.62 ± 0.10	0.60 (0.42, 0.82)	0.06 ± 0.02	0.06 (0.02, 0.12)	0.972 (0.939, 0.984)
CON 60 IR NDM PT/W (N/m.kg)	0.46 ± 0.13	0.43 (0.31, 0.92)	0.52 ± 0.14	0.49 (0.34, 0.99)	0.06 ± 0.02	0.06 (0.02, 0.10)	0.980 (0.953, 0.990)
CON 60 ER DM PT (N/m)	34.00 ± 5.29	34.10 (27.00, 48.40)	38.46 ± 5.37	39.00 (31.00, 52.90)	4.46 ± 1.28	4.53 (1.60, 9.00)	0.946 (0.891, 0.970)
CON 60 ER NDM PT (N/m)	26.07 ± 8.61	22.90 (20.00, 58.00)	30.35 ± 8.79	27.75 (21.90, 63.00)	4.28 ± 1.42	4.25 (0.60, 6.50)	0.908 (0.829, 0.952)
CON 60 ER DM PT/W (N/m.kg)	0.47 ± 0.08	0.45 (0.35, 0.68)	0.53 ± 0.09	0.52 (0.37, 0.74)	0.06 ± 0.02	0.06 (0.02, 0.13)	0.950 (0.893, 0.977)
CON 60 ER NDM PT/W (N/m.kg)	0.36 ± 0.12	0.33 (0.24, 0.81)	0.42 ± 0.13	0.39 (0.29, 0.88)	0.06 ± 0.02	0.06 (0.00, 0.10)	0.922 (0.840, 0.958)
ECC 60 IR DM PT (N/m)	50.02 ± 9.09	48.60 (32.00, 74.56)	53.90 ± 8.89	51.60 (37.89, 77.85)	3.89 ± 1.15	3.80 (1.80, 6.12)	0.985 (0.958, 0.994)
ECC 60 IR NDM PT (N/m)	51.27 ± 9.90	49.70 (31, 70.52)	55.15 ± 10.11	54.35 (33.30, 75.23)	3.88 ± 1.12	4.05 (2.00, 6.40)	0.990 (0.973, 0.996)
ECC 60 IR DM PT/W (N/m.kg)	0.69 ± 0.13	0.71 (0.41, 1.04)	0.75 ± 0.13	0.76 (0.49, 1.08)	0.05 ± 0.02	0.06 (0.02, 0.08)	0.992 (0.978, 0.995)
ECC 60 IR NDM PT/W (N/m.kg)	0.71 ± 0.16	0.71 (0.37, 0.99)	0.77 ± 0.16	0.77 (0.40, 1.06)	0.05 ± 0.02	0.06 (0.02, 0.09)	0.994 (0.984, 0.997)
ECC 60 ER DM PT (N/m)	44.46 ± 10.10	43.40 (25.90, 70.90)	48.20 ± 9.86	47.10 (29.70, 74.40)	3.74 ± 1.26	3.70 (1.30, 6.30)	0.988 (0.967, 0.994)
ECC 60 ER NDM PT (N/m)	45.06 ± 10.19	44.95 (27.60, 65.00)	48.94 ± 10.27	48.10 (30.10, 68.10)	3.88 ± 1.33	4.00 (1.30, 6.30)	0.990 (0.975, 0.996)
ECC 60 ER DM PT/W (N/m.kg)	0.62 ± 0.14	0.61 (0.33, 0.97)	0.67 ± 0.14	0.66 (0.38, 1.02)	0.05 ± 0.02	0.05 (0.02, 0.09)	0.989 (0.969, 0.994)
ECC 60 ER NDM PT/W (N/m.kg)	0.63 ± 0.15	0.62 (0.35, 0.95)	0.68 ± 0.16	0.67 (0.36, 1.02)	0.05 ± 0.02	0.06 (0.01, 0.10)	0.989 (0.973, 0.994)

SNP: Scapular neutral position, MABP: muscle architecture-based position, CON: concentric, ECC: eccentric, IR: internal rotation, ER: external rotation, DM: dominant side, NDM: nondominant side, PT: peak torque, W: weight, N: Newton.

* There was a statistically significant difference between the 2 methods (Wilcoxon signed-rank test p < 0.001),

CI: confidence interval, r_s_: Spearman rho correlation coefficient)

**Table 2:** Test and retest reliability values of the MABP (n = 54).

Measurements	Difference (MABP retest)			ICC (95% CI)	SEM	SDD	SDD%
	Mean ± SD	Median (min, max)	p-value				
CON 60 IR DM PT (N/m)	0.30 ± 1.37	0.69 (−3.40, 2.41)	0.074	0.976 (0.959–0.986)	0.98	2.71	6.10
CON 60 IR NDM PT (N/m)	0.11 ± 1.25	0.13 (−3.54, 2.17)	0.324	0.992 (0.986–0.995)	0.88	2.43	6.49
CON 60 IR DM PT/W (N/m.kg)	0.00 ± 0.02	0.01 (−0.04, 0.03)	0.095	0.982 (0.969–0.99)	0.01	0.04	6.02
CON 60 IR NDM PT/W (N/m.kg)	0.00 ± 0.02	0.00 (−0.04, 0.04)	0.324	0.992 (0.986–0.995)	0.01	0.03	6.57
CON 60 ER DM PT (N/m)	0.03 ± 0.79	−0.24 (−1.20, 2.10)	0.660	0.989 (0.982–0.994)	0.55	1.53	3.98
CON 60 ER NDM PT (N/m.kg)	0.07 ± 0.81	−0.03 (−1.17, 2.00)	0.927	0.996 (0.993–0.998)	0.57	1.58	5.21
CON 60 ER DM PT/W (N/m.kg)	0.00 ± 0.01	0.00 (−0.01, 0.02)	0.721	0.992 (0.987–0.996)	0.01	0.02	3.85
CON 60 ER NDM PT/W (N/m.kg)	0.00 ± 0.01	0.00 (−0.02, 0.03)	0.927	0.996 (0.993–0.998)	0.01	0.02	5.30
ECC 60 IR DM PT (N/m)	0.16 ± 0.78	−0.05 (−1.48, 1.96)	0.360	0.996 (0.993–0.998)	0.55	1.53	2.85
ECC 60 IR NDM PT (N/m)	−0.11 ± 0.83	−0.14 (−1.50, 1.94)	0.137	0.997 (0.994–0.998)	0.58	1.61	2.92
ECC 60 IR DM PT/W (N/m.kg)	0.00 ± 0.01	0.00 (−0.02, 0.03)	0.377	0.997 (0.994–0.998)	0.01	0.02	2.84
ECC 60 IR NDM PT/W (N/m.kg)	0.00 ± 0.01	0.00 (−0.02, 0.03)	0.150	0.997 (0.996–0.999)	0.01	0.02	2.91
ECC 60 ER DM PT (N/m)	0.08 ± 0.92	−0.02 (−2.79, 2.50)	0.698	0.996 (0.993–0.998)	0.64	1.79	3.71
ECC 60 ER NDM PT (N/m)	0.15 ± 0.72	0.10 (−2.08, 1.82)	0.123	0.997 (0.996–0.998)	0.52	1.43	2.93
ECC 60 ER DM PT/W (N/m.kg)	0.00 ± 0.01	0.00 (−0.04, 0.03)	0.662	0.996 (0.993–0.998)	0.01	0.02	3.67
ECC 60 ER NDM PT/W (N/m.kg)	0.00 ± 0.01	0.00 (−0.03, 0.03)	0.121	0.998 (0.996–0.999)	0.01	0.02	3.00

ICC: Intraclass correlation coefficient (higher than 0.90 was considered excellent reliability), SEM: standard error of measurement, SDD: smallest detectable difference.
